# Osteomyelitis of the pubic symphysis caused by methicillin-resistant *Staphylococcus aureus* after vaginal delivery: a case report and literature review

**DOI:** 10.1186/s12879-019-4595-x

**Published:** 2019-11-08

**Authors:** Stefano Cosma, Fulvio Borella, Andrea Carosso, Agata Ingala, Federica Fassio, Tiziana Robba, Aldo Maina, Luca Bertero, Chiara Benedetto

**Affiliations:** 1 Gynecology and Obstetrics 1, Department of Surgical Sciences, City of Health and Science, University of Torino, Torino, Italy; 2 Gynecology and Obstetrics 2, Department of Surgical Sciences, City of Health and Science, University of Torino, Torino, Italy; 3 Department of Radiology, City of Health and Science, CTO Hospital, Torino, Italy; 4 General Medicine Unit, City of Health and Science, Sant’Anna Hospital, Torino, Italy; 5 Department of Medical Sciences, City of Health and Science, University of Torino, Torino, Italy

**Keywords:** Pregnancy, Postpartum, Pubic diastasis, Septic arthritis, Osteomyelitis, *Staphylococcus aureus*

## Abstract

**Background:**

Osteomyelitis of the pubic symphysis is a rare cause of pelvic pain after delivery, mainly caused by *Staphylococcus aureus* and *Pseudomonas aeruginosa*.

The clinical context is the same as the more common diastasis of the pubic bone, but the presence of intense local pain in association with fever should prompt further clinical work-up based on blood chemistry, microbiology and diagnostic imaging. We report the first case of methicillin-resistant *Staphylococcus aureus* osteomyelitis of the pubic symphysis occuring after the delivery.

**Case presentation:**

A 39-year-old woman developed pain over the pubic bone 12 h after the delivery. After 72 h fever rose and laboratory examination showed elevation of C-reactive protein and procalcitonin levels. Pelvic x-rays and magnetic resonance showed pubic diastasis, joint effusion, tiny irregularities of articular surfaces and, severe bone edema. The patient was started on broad spectrum intravenous (IV) antibiotics (piperacillin-tazobactam) and then replaced to IV vancomycin and oral levofloxacin based on antibiogram result. She was then discharged with oral antibiotic therapy and fully recovered.

**Conclusions:**

Due to the rarity of this disease, we compared our experience with the other cases of osteomyelitis of pubic symphysis occurring in peri-postpartum reported in the literature. The course of osteomyelitis was favourable in all patients, and only in one case an additional orthopedic procedure for symphysis fixation was necessary. Knowledge of this rare condition is important to enable prompt diagnosis and treatment.

## Background

Osteomyelitis of the pubic symphysis is a rare infectious disease associated with local bone destruction and frequent involvement of the joint (septic arthritis).

This infection is commonly caused by the opportunistic bacteria *Staphylococcus aureus* (*S. aureus*) and *Pseudomonas aeruginosa* (*P. aeruginosa*). Typical clinical features are pubic and groin pain, pubic tenderness, fever > 38 °C, leukocytes > 11 × 10^9^/L, and bacteremia. The main risk factors are previous female incontinence surgery, intense physical activity, pelvic malignancy, and intravenous drugs abuse, however, septic arthritis of the pubic symphysis may exceptionally occur as a complication of the postpartum [1–3]. Persistent postpartum pubic pain is quite common and may be caused by diastasis of pubic bone with the widening of the symphysis from 4 to 5 mm (normal value) up to 5 cm. The peripartum pubic separation occurs from 1 in 300 to 1 in 30,000 deliveries [4]. Septic arthritis of the same site is an extremely rare cause of postpartum pelvic pain and only a few cases have been reported in the medical literature so far [5–11]. We report the case of a 39-year-old woman who developed an osteomyelitis and septic arthritis of pubic symphysis caused by methicillin-resistant *S. aureus* (MRSA) after a normal vaginal delivery and compare our experience with other cases previously reported. Informed, written consent was received from the patient for publication.

## Case presentation

A 39-year-old pregnanat (para 3) at 41 weeks of gestation was admitted to Sant’Anna Hospital with active labor. Her clinical history was notable for gestational diabetes mellitus. At 35 weeks of gestation, the patient performed a vaginal swab for group B streptococcus screening, and was negative. She delivered a macrosomic fetus of 4530 g without complications during the labor.

**Fig. 1:**
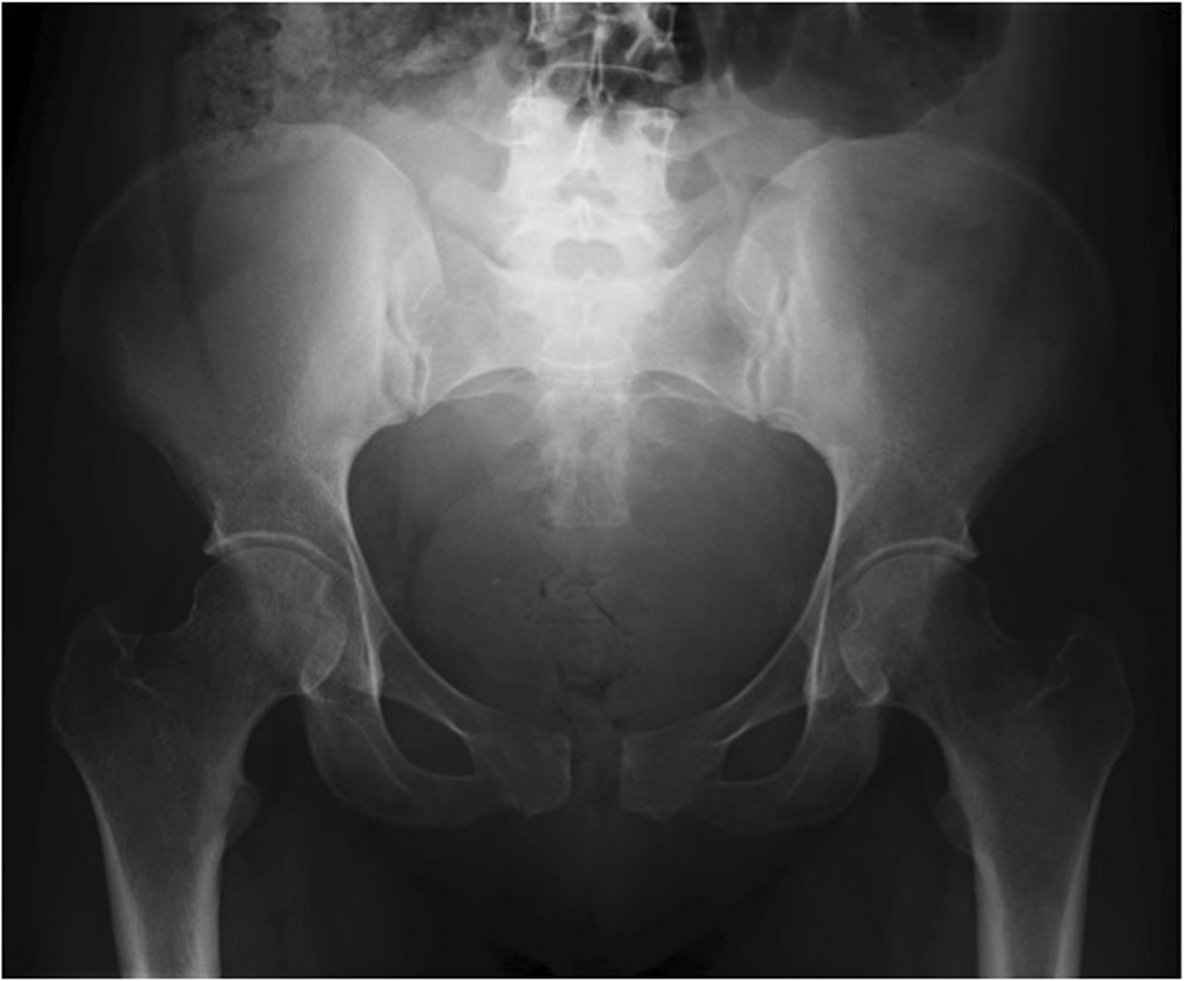
Plain X-ray of the pelvis performed at day 4. The antero-posterior pelvic radiograph reveals pubic symphysis diastasis and tiny irregularities of articular surfaces

**Fig. 2:**
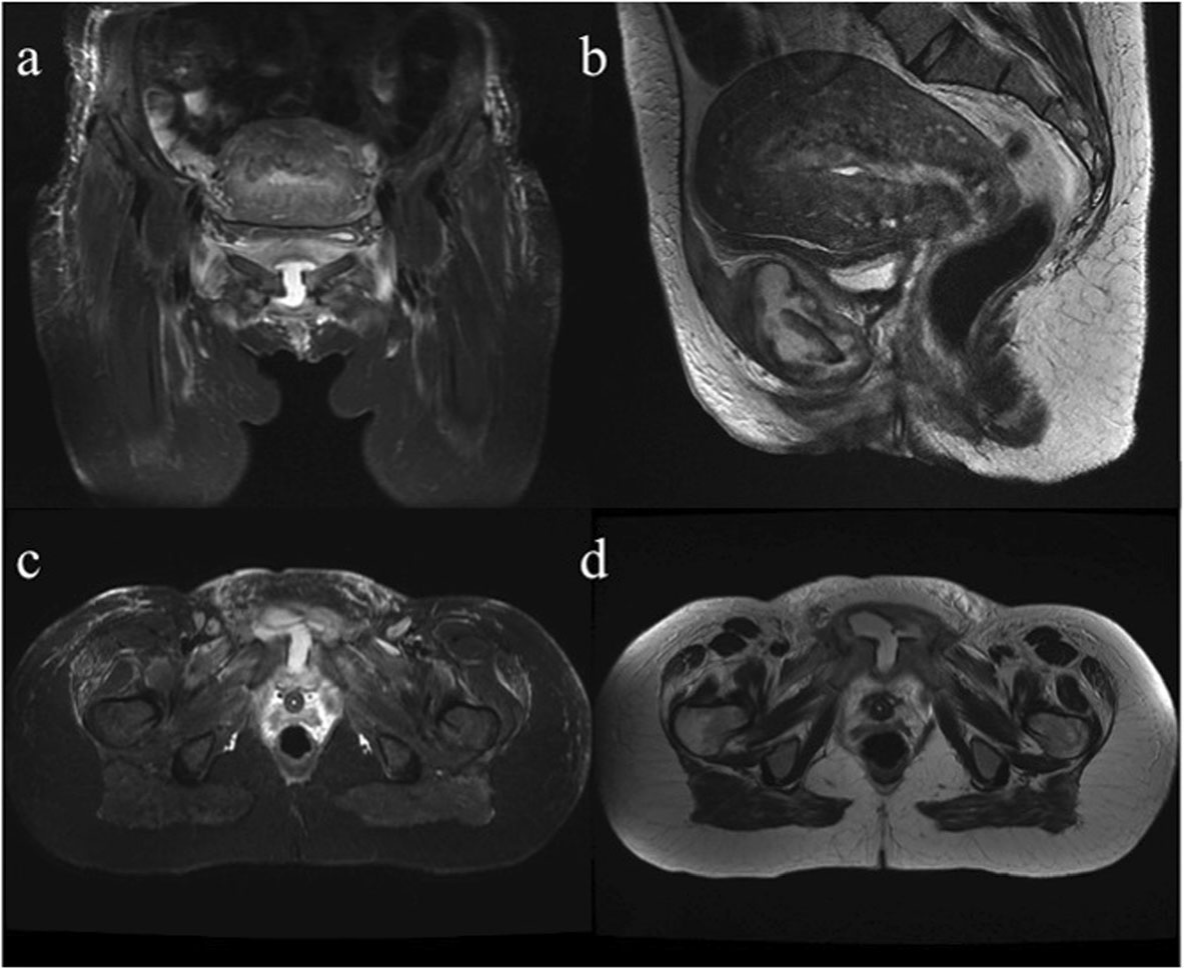
Pelvic magnetic resonance performed at day 8. Coronal short TI inversion recovery (STIR) (**a**), sagittal T2-weighted (**b**), axial STIR (**c**), and axial T2-weighted (**d**) images show pubic symphysis enlargement, abundant joint effusion with synovial thickening forming a pseudo-capsulated fluid collection within the symphysis, severe bone edema involving both pubic branches and edematous subcutaneous tissues

Twelve hours later she complained of increasing pain over the pubic bone radiating to the groin. She could not stand or walk and any active or passive movement of thighs and hips (flexion/extension and adduction/abduction) evoked intense regional pain. Her symptoms were thought to be related to pelvic girdle strain during delivery. After 72 h her temperature rose to 39.5 C°, while her clinical examination was otherwise unremarkable. Laboratory investigation revealed leukocytosis (21 × 10^9^/L, reference range [ref] 4–10 × 10^9^/L; 85.3% neutrophils) and elevation of C-reactive protein (CRP) levels (379.0 mg/L, ref. 5.0 mg/L) raised the suspicion of an undetected infection. Her serum procalcitonin (PCT) was elevated (4.0 ng/mL, ref.: negative < 0.5 ng/ml, gray zone 0.5–2.0 ng/ml, positive > 2.0 ng/ml). Blood culture was performed and the patient was started on broad spectrum intravenous (IV) antibiotics (piperacillin-tazobactam 4.5 g every 6 h). At the same time, transperineal ultrasound evaluation showed a round-shaped hypoechogenic, fluid-filled cavity between the articular surfaces of the pubis. At day 4 a plain X-ray showed a pubic diastasis of 2 cm and tiny irregularities of articular surfaces (Fig. 1). The blood cultures were positive for Gram-positive cocci, later identified as MRSA, so the antibiotic regimen was changed to IV vancomycin (1 g twice daily) and oral levofloxacin (500 mg once a day), this choice was based on the result of antibiogram. A screening for MRSA was also performed on the newborn and was negative. At day 8, the patient underwent a pelvic magnetic resonance imaging (MRI) showing symphysis enlargement, abundant joint effusion with synovial thickening forming a pseudo-capsulated fluid collection within the symphysis, severe bone edema involving both pubic branches and edematous subcutaneous tissues (Fig. 2). These clinical and radiological findings were highly suggestive of acute osteomyelitis and septic arthritis. The patient improved after 2 weeks and then IV vancomycin was stopped. She was discharged after 18 days of hospitalization and continued oral levofloxacin (500 mg gr once a day) plus rifampicin (600 mg once a day). Finally, the antibiotic therapy was stopped after 4 weeks; the patient was afebrile with normal blood tests and she did not report local pubic pain or functional limitations. Nine months after delivery the patient was still asymptomatic and pelvic MRI revealed complete fluid reabsorption at the pubic symphysis.

## Discussion and conclusions

A literature search was conducted using PubMed, EMBASE and Google Scholar. For this purpose, we used the following key words “osteomyelitis pubic symphysis”, “septic arthritis pubic symphysis”, “pubic symphysitis”, “pubic osteitis”, “pregnancy” “postpartum” and “delivery”. With the inclusion of our patient, only 9 cases of osteomyelitis of the pubic symphysis in peri/postpartum have been reported in the literature and detailed information regarding these cases are reported in Table 1 [5–11].

**Table 1:** Documented cases reports of osteomyelitis of the pubic symphysis in peri/post-partum

Reference	Age	Parity	Type of delivery	Clinical features	Radiological finding	Pathogen	Treatment	Outcomes
Eskridge et al. [5]	33 years	1	Vaginal delivery with shoulder dystocia required episiotomy, suprapubic pressure and McRobert’s maneuver	Pubic pain with ambulation starting 1 day after delivery; fever; leukocytosis	Diastasis of the symphysis pubis, erosion of pubic rami, vulvar edema	*Staphylococcus epidermidis* *Enterococcus faecalis*	Intravenous (IV) ampicillin, sulbactam sodium changed to cefazolin and metronidazole Surgical debridemen	Complete recovery
Lovisetti et al. [6]	25 years	2	Normal vaginaldelivery	Pubic pain starting 1 day after delivery; fever; ↑ erythrocyte sedimentation rate (ESR), leukocytosis	Diastasis of the symphysis \pubis, bone rarefaction, suprapubic abscess	*Staphylococcus aureus*	IV Mezlocillin, netilmicin Surgical debridement Stabilisation of the symphysis with a Hoffmann external frame	Complete recovery
Gamble et al. [7]	37 years	1	Cesarean section	Pubic pain with ambulation starting at 28 weeks of gestation; erythema and edema over the labia, mons pubis, and lower abdomen; low grade fever; normal complete blood count	Diastasis of the symphysis pubis, enhancing mass with a high T1 signal suggesting superinfected hematoma or abscess	*Staphylococcus aureus* *Pseudomonas aeuroginosa* *Serratia marcescens*	IV vancomycin, levofloxacin, metronidazole Surgical debridement	Complete recovery
Ikpeme et al. [8]	28 years	Not reported	Normal vaginal delivery	Pubic pain starting 8 weeks after delivery; low grade fever; ↑ ESR, normal complete blood count	Diastasis of the symphysis pubis, irregular erosion, lytic lesions	*Pseudomonas aeuroginosa*	Antibiotics (not specified) Surgical debridement	Complete recovery
Dunk et al. [9]	43 years	Not reported	Normal vaginal delivery	Pubic and groin pain starting 5 days after delivery, low grade fever, leukocytosis, ↑ C-protein reactive (CPR)	Reactive sclerosis, rarefaction, osteolysis and joint irregularity	*Streptococcus* group G	IV cefuroxime, metronidazole Surgical debridement	Complete recovery
	31 years	Not reported	Normal vaginal delivery	Pubic pain starting 12 h after delivery, fever, leukocytosis, ↑ CPR	Reactive sclerosis, rarefaction, osteolysis and joint irregularity	*Staphylococcus epidermidis*	IV cefuroxime	Complete recovery
Lawford et al. [10]	27 years	1	Emergency cesarean section for fetal tachycardia with decreased variability and late decelerations to the cardiotocography	Groin pain, tenderness to palpation in the right iliac fossa with signs of peritonism, marked vulvar oedema and fever starting at 37 weeks of gestation; normal complete blood count, ↑ CPR	5 cm collection surrounding the symphysis pubis with extension into the soft tissues	*Staphylococcus aureus*	IV ceftriaxone, metronidazole and gentamicin changed to flucloxacillin and cephazolin based on antibiogram results	Complete recovery
Froberg et al. [11]	33 years	Not reported	Normal vaginal delivery	Pubic pain starting at 12 weeks of gestation; normal complete blood count	Fluid within the symphysis, bilateral oedema of the pubic rami and bony erosions.	*Staphylococcus capitis Cutibacterium acnes*	IV clindamycin	Complete recovery
Current case report	39 years	3	Normal vaginal delivery	Pubic pain starting 12 hours after the delivery; fever, leukocytosis, ↑ CPR, ↑ procalcitonin	Diastasis of the symphysis pubis, tiny irregularities of articular surfaces, pseudo-capsulated fluid collection within the symphysis, joint effusion	Methicillin-resistant *Staphylococcus aureus*	IV piperacillin-tazobactam changed to vancomycin and levofloxacin based on antibiogram, then after 2 weeks, oral combination of levofloxacin plus rifampicin.	Complete recovery

No particular obstetric risk factors were reported for this disease: most cases were preceded by normal vaginal delivery. Only in one case the delivery was complicated by shoulder dystocia [5] and in two cases the osteomyelitis of pubic symphysis occurred after cesarean section [7, 10]. The pathogenesis of this rare peripartum complication is unknown. In one case, the onset of pubic pain was concurrent with submandibular cellulitis and the authors suggested a hematogenous origin of the pubic infection [5].

The most common pathogens involved in osteomyelitis were *S. aureus* and *P. aeurginosa,* a trend further confirmed by our case of osteomyelitis of pubic symphysis [6–8, 10]. Other pathogens reported in peri/postpartum were *Serratia marcescens, Streptococcus* group G, *Cutibacterium acnes, Staphylococcus capitis*, *Staphylococcus epidermidis* and *Enterococcus faecalis* [5–11]*.* In our and previously reported cases of osteomyelitis of pubic symphysis, the culture tests were always positive for a pathogen while in 4 cases the total blood count was normal [7, 8, 10, 11].

All patients were treated with antibiotic therapy and only in 5 cases a surgical debridement was performed [5–9]. No patient had sequelae after the treatment and only in one case stabilization of the pubic symphysis by an external frame was required [6]. Also in our case, antibiotic therapy alone was sufficient to achive a successful outcome.

Postpartum pubic pain is a common and self-limiting disorder requiring only symptomatic therapy in most cases. The distinction between the more common diastasis and osteomyelitis of the pubic symphysis is critical for prognosis and treatment; moreover, non-infective pubis osteitis can occur after delivery, which can cause mild fever and has similar X-ray findings to those of osteomyelitis creating a potential diagnostic pitfall [2]. Local pain, inability to walk and fever are suggestive of an inflammatory/infective process, but the clinical suspicion should be confirmed by laboratory data, blood and/or local fluid cultures and imaging findings.

Puerperal endometritis, characterised by pelvic pain, uterine or parametrial tenderness, maternal tachycardia, foul-smelling lochia or maternal leukocytosis (> 12 × 10^9^/L), should also be considered in the differential diagnosis [12]. Regarding laboratory tests, in acute staphylococcal osteomyelitis, about 40% of patients presented with only a moderate leukocytosis count of (10.5 × 10^9^/L considering a range of 4.5 to 11 × 10^9^/L) and in up to 40% of cases, microbiological tests produced false-negative results [3]. In 4 out of the 9 reported osteomyelitis of pubic symphysis cases, a normal blood count was observed, but culture tests resulted consistently positive.

Possibly, in predisposed subjects, the pelvic soft tissues trauma occurring during vaginal delivery or cesarean section can promote the colonization and the contiguous spread of some bacterial strains, moreover, the colonization of the genital tract in pregnancy by MSRA, with the risk of vertical transmission, has been described in the literature [13, 14].

In one case, the onset of pubic pain was concurrent with submandibular cellulitis suggest a hematogenous origin of the pubic infection [5].

Of note, some authors [15, 16] also suggest to perform a screening of high-risk newborns for MRSA colonization or infection in order to promptly implement preventive measures.

Determination of serum PCT had been reported in a large series of osteomyelitis/septic arthritis, however the value often falls within a” gray zone” and false-negative results do occur, suggesting the use of a lower cut-off value to define positivity in this setting [17], however this marker may be more valuable than CRP for the diagnosis of this infectious disease [18], Paccolat et al evaluated PCT levels in pregnant women with no signs of clinical infection at different gestational ages; the authors suggest using a cut-off PCT level of 0.25 ng/mL to rule out infection during the third trimester, at delivery, and at the immediate postpartum [19], this is the first case of osteomyelitis of pubic symphysis in postpartum in which the PCT proved to be an useful infection marker.

Diagnostic imaging plays a crucial role in providing a correct framework for differential diagnosis of pubic pain. Transperineal or suprapubic sonography may be useful to reveal symphysis diastasis and show fluid collections, also enabling an ultrasound-guided needle aspiration for fluid aspiration or open bone biopsy with histopathologic examination and culture which are the gold standard for the microbiologic diagnosis of osteomyelitis. In our case, fluid aspiration was deferred because blood cultures were positive and consistent with radiologic findings. X-ray allows detecting symphysis diastasis, juxta-articular osteoporosis and bony erosions, but only in an advanced stage. Delayed signs of septic arthritis could be sclerosis, osteophytosis, and progressive joint destruction. The same abnormalities may be identified through computed tomography achieving a better evaluation of bony erosions, surface irregularities, abscesses and symphyseal diastasis [20]. However, magnetic resonance is the most sensitive tool and it allows early detection of suspicious findings for septic arthritis such as synovial enhancement, perisynovial edema, and joint effusion. Specificity, however, is not high since it is difficult to distinguish between reactive edema and osteomyelitis [20]. MRI also enables the identification of other possible causes of pubic pain due to postpartum complications such as pelvic hematoma or abscess, genitourinary injuries, and insufficiency fractures [21]. Furthermore, MRI findings are helpful to assess the response to treatments, especially the amount of joint effusion and the presence of abscess are probably the most reliable factors for monitoring therapy efficacy in osteomyelitis/septic arthritis [22].

Based on the reported cases, outcome seems to be excellent in most cases if prompt treatment is established. Regarding the specific regimen for MRSA osteomyelitis, optimal duration of antibiotic therapy is still a topic of debate: the clinical guidelines by the Infectious Disease Society of America for the treatment of MRSA infection suggest a minimum of 8 weeks for osteomyelitis and a 3–4-week of therapy for septhic arthritis, but a precise duration of the antibiotic therapy has not been clearly defined [3, 23, 24].

In conclusion, postpartum osteomyelitis of pubic symphysis is a rare disorder, which should be considered in women presenting with increasing pubic pain that does not respond to painkillers. Broad spectrum antibiotics are the first line treatment and must be administered while culture tests are ongoing. Surgical debridement may be considered in case of non-response to antibiotics; nevertheless, in staphylococcal osteomyelitis, relapse can occur in up to 40% of cases after surgical debridement [3]. Knowledge of this rare possible complication is necessary to enable prompt diagnosis and timely treatment.

## Data Availability

All data analysed during this study are included in this published article.

## Abbreviations

CRP: C-reactive protein
ESR: Erythrocyte sedimentation rate
IV: Intravenous
MRI: Magnetic resonance imaging
MRSA: Methicillin-resistant *S. aureus*
PCT: Procalcitonin
Ref: Reference range
